# Therapeutic Potential of Wogonin–Aloperine Co-Amorphous for Oral Squamous Cell Carcinoma

**DOI:** 10.3390/pharmaceutics17091204

**Published:** 2025-09-16

**Authors:** Guoliang Wu, Han Li, Zhongshui Xie, Song Ni, Yiming Zhu, Chunxue Jia, Chenyu Pan, Shaoyan Liu, Hongjuan Wang

**Affiliations:** 1 School of Chinese Materia Medica, Beijing University of Chinese Medicine, Beijing 100102, China; 15735010317@163.com (Z.X.); a15242876702@163.com (C.J.); xbpcyatx@163.com (C.P.); 2 Department of Head and Neck Surgery, National Cancer Center/National Clinical Research Center for Cancer/Cancer Hospital, Chinese Academy of Medical Sciences and Peking Union Medical College, Beijing 100021, China; wugl_cicams@outlook.com (G.W.); lihanw11@163.com (H.L.); nisong168@sina.com (S.N.); drymzhu@163.com (Y.Z.)

**Keywords:** oral squamous cell carcinoma, patient-derived xenograft model, wogonin–aloperine co-amorphous

## Abstract

**Background**: Oral squamous cell carcinoma (OSCC) is a major epithelial malignancy of the head and neck with high morbidity and mortality. The conventional antineoplastic medications used in clinical practice have become less effective due to the heterogeneity of tumors, accompanied by severe side effects. Therefore, the development of novel chemotherapeutic agents has become an important goal of anti-OSCC therapy. **Methods**: Our group has previously developed a novel wogonin–aloperine co-amorphous (Wog–Alop). In this study, the anti-OSCC efficacy of Wog–Alop was evaluated by a patient-derived tumor xenograft (PDX) model. Subsequently, network pharmacology was employed to predict the key targets of Wog–Alop on OSCC, and the predicted key targets were further confirmed by Western blot and immunochemistry. **Results**: The results revealed that Wog–Alop manifests the higher efficacy in inhibition of OSCC proliferation by regulating the expression of the key targets, Bcl-2, Bax, P53, and Caspase3, implying that the apoptotic mechanism is implicated in Wog–Alop-induced inhibition of proliferation in OSCC. **Conclusions**: Collectively, the present work demonstrated anti-OSCC bioactivity of Wog–Alop, suggesting that Wog–Alop could be developed as an innovative therapeutic agent for OSCC therapy.

## 1. Introduction

Oral squamous cell carcinoma (OSCC) is a major epithelial malignancy of the head and neck, ranking as the sixth leading malignant tumor worldwide [1]. As documented in the latest GLOBOCAN estimates (2022), OSCC accounts for 389,846 new cases globally (2.0% of total cancers) and 188,438 deaths (1.9% of cancer mortality), reflecting a 3.2% rise in incidence and 6.0% increase in mortality compared to the 2020 estimates. Despite great progress in diagnosis and therapy, the 5-year survival rate of patients with OSCC has not improved significantly in the last few decades [2]. Currently, the conventional surgical treatment often fails to achieve a complete cure, especially for late-stage metastatic OSCC, and radiation and chemotherapeutic agents used in clinical practice are frequently associated with undesirable adverse events [3,4,5]. Moreover, the biological complexity of OSCC limits the efficacy of a single chemo-therapeutic agent [6,7,8]. Natural plants and plant-derived formulations are highly efficacious in the treatment of cancer and have been used for cancer treatment from ancient times. For example, plant-derived anticancer agents such as paclitaxel [9,10], camptothecin [11,12], and vincristine [13,14] have achieved widespread clinical applications due to their demonstrated antitumor efficacy.

Inspired by the therapeutic efficacy of natural products, many investigations focused on the therapeutic potential of various secondary metabolites present in plants and found that flavonoids exhibit excellent antitumor activities [15,16] by regulating multiple pathways with less toxicity [17,18,19]. Wogonin, a pharmacologically active flavonoid derived from *Scutellaria baicalensis Georgi*, has demonstrated remarkable therapeutic efficacy against multiple malignancies, including head and neck cancers [20], breast cancer [21], ovarian cancer [22], and lung cancer [23], while maintaining cytoprotective properties toward non-transformed cells, thereby circumventing the dose-limiting toxicities associated with conventional chemotherapeutics. The antitumor mechanisms of wogonin encompass both antiproliferative activity and activation of programmed cell death pathways, including apoptosis and autophagy, across diverse tumor cell lineages [24,25]. In addition to its standalone inhibitory effect on tumor cell proliferation, wogonin has a sensitizing effect on conventional chemotherapy drugs and can even reverse drug resistance [26], which makes it a promising candidate for a chemotherapeutic agent. However, its poor water solubility [27] leads to low bioavailability and limits its further exploitation.

Our group has previously developed wogonin–aloperine co-amorphous (Wog-Alop) drug delivery systems and significantly improved the bioavailability of wogonin [28]. At present work, the antitumor effect of Wog–Alop against OSCC in vivo was further evaluated by a patient-derived tumor xenograft (PDX) model, and the results indicated that Wog–Alop demonstrates higher efficacy in inhibiting the progression of OSCC than wogonin, which could be reasonably explained by the fact that the improved bioavailability contributes to the improvement of efficacy in anti-OSCC. Notably, PDX models retain key characteristics of primary patient tumors, including gene-expression profiles and drug responses, which makes them reliable in vivo models for evaluating chemotherapy drugs. To elucidate the molecular mechanisms underlying Wog–Alop treatment, network pharmacology was employed to predict the possible targets in OSCC. Subsequently, the predicted key targets, Bcl-2, Bax, P53, and Caspase3 were confirmed in OSCC tissue by Western blot and immunohistochemistry, characterized by the upregulation of tumor suppressor p53 and pro-apoptotic Bax, downregulation of anti-apoptotic Bcl-2, and subsequent caspase-3 activation. Taken together, these findings position Wog–Alop as a promising candidate for development into an innovative strategy in OSCC management.

## 2. Materials and Methods

### 2.1. Reagents and Materials

Wogonin (Wog) and aloperine (Alop) were bought from Yuanye Biotechnology Co., Ltd. (Shanghai, China) with purity greater than 98%. Chromatographic grade methanol, acetonitrile, and formic acid solvents were obtained from Thermo Fisher Scientific Co., Ltd. (Waltham, MA, USA). Physiological sodium chloride solution was bought from Kelun Pharmaceutical Co., Ltd. (Chengdu, China). Primary antibodies and HRP-labeled anti-goat and anti-rabbit second antibodies were purchased from Servicebio Technology Co., Ltd. (Wuhan, China). 

### 2.2. Animals

The six-week-old BALB/c nude mice were purchased from Beijing Vital River Laboratory Animal Technology Co., Ltd. (Beijing, China) weighing 20 ± 2 g, and were randomly divided into cages (6 rats per cage) and acclimatized for at least one week with free access to food and water. The experimental protocols were in accordance with institutional guidelines for the care and use of laboratory animals of National Clinical Research Center for cancer/Cancer Hospital, Chinese Academy of Medical Science and Peking Union Medical College (NCC2023A237).

### 2.3. In Vivo Anti-OSCC Efficacy of Wog–Alop

After acclimatizing to laboratory conditions for one week, the BALB/c nude mice were surgically implanted with tumor tissue 1 mm in diameter from OSCC patients to create PDX model. As the tumor volume exceeds 100 mm^3^, the nude mice were randomly divided into four groups (*n* = 6): Wog–Alop, Wog, Alop, and control group, administered by intraperitoneal injections with Wog–Alop (150 mg/kg), Wog(80 mg/Kg), and Alop (70 mg/kg), respectively, and the control was only injected with an equal dose of 0.9% saline once a day. The tumors were measured every other day and the volume of tumors was calculated by the formula: (length × width^2^)/2 [29]. All experimental procedures were conducted in accordance with institutional animal care and use committee protocols. The nude mice were monitored until the tumors reached 15 mm in diameter, at which they were humanely euthanized according to humane endpoints, and their tumors, heart, liver, kidneys, lungs, and spleen were harvested for histopathological examination.

### 2.4. Network Pharmacology

The potential targets of wogonin were predicted by Traditional Chinese Medicine Systems Pharmacology Database (TCMSP, https://old.tcmsp-e.com/tcmsp.php) [30], Swiss Target Prediction (http://swisstargetprediction.ch/) [31], Super Pred (https://prediction.charite.de/) [32] and Pharm Mapper (https://lilab-ecust.cn/pharmmapper/index.html) [33], and the results after merging and removing duplicates were denoted as potential targets of Wog–Alop. The OSCC-related targets were collected in Genecards (https://www.genecards.org/) [34] and Online Mendelian Inheritance in Man (OMIM, https://www.omim.org/) using the keyword “oral squamous cell carcinoma”. The intersection targets were obtained by intersecting potential targets of Wog–Alop with the OSCC-related targets through Draw Venn Diagram (https://bioinformatics.psb.ugent.be/webtools/Venn/). Protein–protein interaction (PPI) analysis of the intersection targets was performed through the String (https://cn.string-db.org/). Gene Ontology (GO) and Kyoto Encyclopedia of Genes and Genomes (KEGG) pathway enrichment analysis of intersection targets were performed by Metascape (https://www.metascape.org). The pathways and corresponding targets of the KEGG pathway enrichment analysis results were imported into cytoscape 3.7.1 software (access occurred on 20 June 2024) to construct and visualize the target-pathway network.

### 2.5. Molecular Docking and Dynamics Simulations

The molecular docking was performed with Discovery Studio 4.5 software (access occurred on 25 June 2024). The structure of Wog–Alop was drawn by KingDraw (access occurred on 22 June 2024), and then the crystal structures of P53, Bax, Caspase3, and Bcl-2 proteins were obtained from Protein Data Bank (PDB) database (https://www.rcsb.org/) (access occurred on 22 June 2024) [35]. Excess sequence, water, and ligands were removed from the protein crystal structures. After that, the chemical structure of the Wog–Alop was set as a ligand and CHARMM general force field was added. Subsequently, CDOCKER DOCK (access occurred on 25 June 2024) was performed to save 2D and 3D visualization plots, and binding energy between wogonin, aloperine, Wog–Alop and target proteins were calculated. To test the stability of the protein-ligand complexes obtained after molecular docking, protein-ligand complexes were pre-processed and the CHARMM 36 force field was added to the processed protein structure, followed by molecular dynamic simulation in the Standard Dynamics Cascade module. And the root means square deviation (RMSD), root means square fluctuation (RMSF), and number of hydrogen bonds in the molecular dynamic simulation trajectories were calculated by the Analyze Trajectory (access occurred on 25 June 2024).

### 2.6. Western Blots Analysis

Fresh tumor tissues were cut into small pieces and placed in a homogenization tube after washing with pre-cooled PBS. Lysis buffer with protease inhibitor was added, and homogenization was carried out, centrifuging to collect the supernatant. Subsequently, the cells were collected and incubated with ice-cold lysis buffer for 30 min on ice before being centrifuged at 4 °C at 12,000 r/min for 5 min. The lysate protein (10 μg) was transferred onto polyvinylidene difluoride membranes and electrophoretically separated by 15% SDS-PAGE. After blocking with 5% non-fat milk, membranes were incubated with primary antibodies overnight at 4 °C. After rinsing with TBST, membranes were incubated with secondary antibody conjugated with HRP at room temperature for 30 min. The blots were examined using a AIWBwellTM (Servicebio Technology Co., Ltd., Wuhan, China). The relative expression of proteins was quantified using highly sensitive ECL western blotting substrate. 

### 2.7. Immunohistochemistry Staining

Firstly, paraffin-embedded tumor tissue sections were dewaxed with xylene and then hydrated in anhydrous ethanol. Subsequently, antigen retrieval was conducted by immersing the sections in pH 9.0 EDTA with medium heat for 8 min by microwave, followed by blocking endogenous peroxidase with 3% solution of hydrogen peroxide for 25 min. After blocking with 3% bovine serum albumin for 30 min, the sections were incubated with primary antibodies at 4 °C overnight. Washing with PBS, the sections were probed with the desired secondary antibody for 50 min. Finally, the sections were staining by diamino-benzidine staining solution for visualization at room temperature. The antibodies are listed in Table S1.

### 2.8. TUNEL Staining

Briefly, the dewaxing tumor tissue sections were incubated in 3% hydrogen peroxide in methanol for 5 min to block endogenous peroxidase activity, and then repaired with proteinase K, followed by incubating with permeabilization wash solution (0.1% triton) for 5 min at room temperature. After covering with a buffer for equilibrium for 10 min, the tissue sections were then treated by terminal deoxynucleotidyl transferase (TdT) enzymes, dUTP and buffer with a ratio of 1:5:50 and incubated for 2 h at 37 °C. Subsequently, the tissue sections were washed with PBS (pH 7.4) for 5 min while adding DAPI dyeing fluid and incubated for 10 min. Finally, the tissue sections were placed in PBS (pH 7.4) for decolorization and sealed with anti-fluorescent quenching seal. The tissue sections were checked with a NIKON ECLIPSE C1 microscope (Nikon Corporation, Tokyo, Japan), and the images were collected using a NIKON DS-U3(Nikon Corporation, Tokyo, Japan).

### 2.9. Statistical Analysis

The data were statistically compared using GraphPad Prism 9.5 (access occurred on 5 May 2025). The values were presented as mean ± standard deviation (SD) and the statistically significant difference between groups was evaluated by a two-way ANOVA test. The significance of difference is indicated when * *p* < 0.05 or ** *p* < 0.01.

## 3. Results

### 3.1. In Vivo Anti-OSCC Efficacy of Wog–Alop

**Figure 1:**
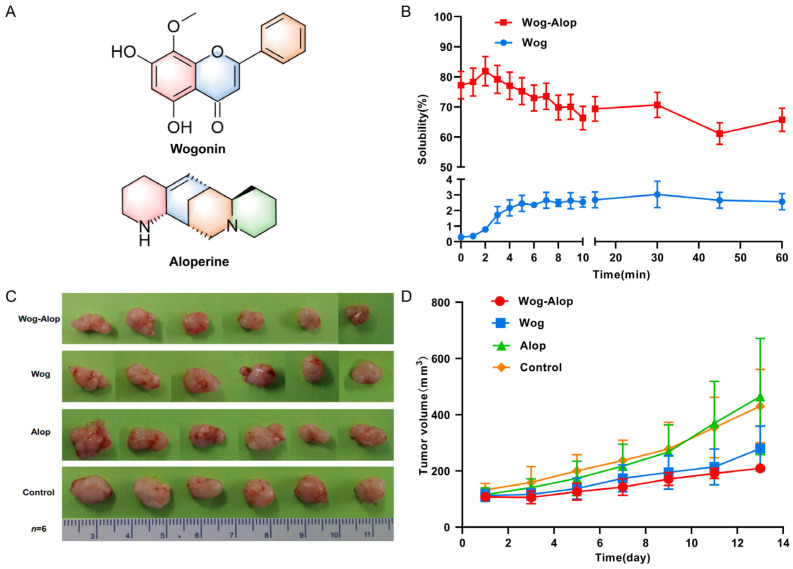
In vitro solubility and in vivo effect on the inhibition of tumor growth of wogonin and Wog–Alop. (**A**) The structure of wogonin and aloperine; (**B**) The solubility percent of wogonin and Wog–Alop in water; (**C**) The image of the resected OSCC tumor from nude mice; (**D**)Tumor volumes of control, aloperine, wogonin and Wog–Alop (*n* = 6).

The inhibition of tumor growth by Wog–Alop on OSCC in vivo was investigated by a PDX model. As shown in Figure 1, the proliferation of OSCC was evidently inhibited by wogonin and Wog–Alop, whereas the tumor-suppressing effect of Wog–Alop was better than wogonin, which can be explained by the fact that the improved solubility of Wog leads to a better bioavailability, thereby enhancing the anti-OSCC effects. Meanwhile, it can be clearly seen that the tumor volume of the Wog–Alop group is smaller than that of the other three groups after two weeks of treatment. The results showed that, as a novel lead compound, the Wog–Alop demonstrates effective inhibition of OSCC and prolongs the survival of the mice with no observed organ toxicity (Figure S1). In conclusion, the present work confirmed the favorable anti-OSCC efficacy of Wog–Alop in vivo, providing a solid foundation for the design of clinical trials to test the Wog–Alop in human OSCC. Further experiments need to be conducted to explore the mechanism of action in exerting its anti-OSCC effects, including the inhibition of tumor cell proliferation, induction of apoptosis, and suppression of angiogenesis.

### 3.2. Network Pharmacology

**Figure 2:**
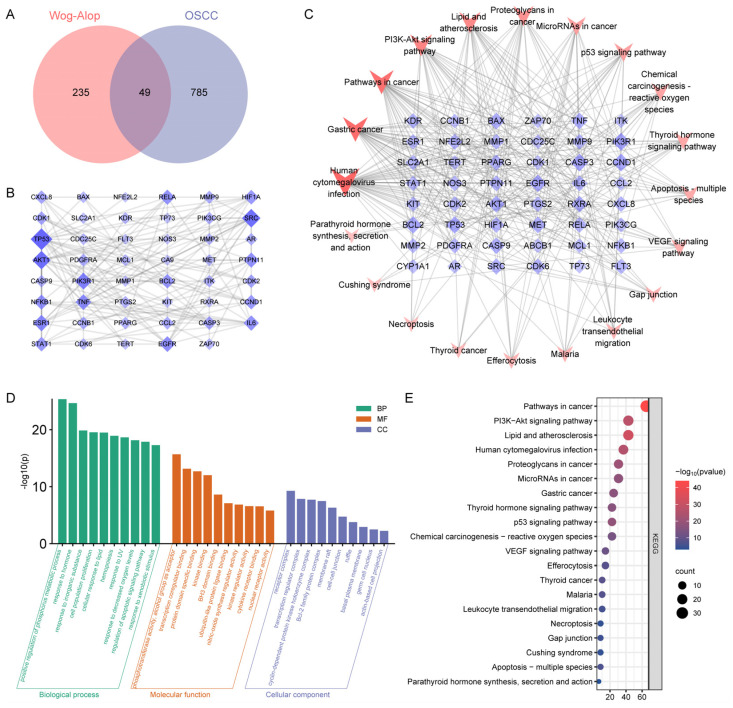
Exploration of key targets of Wog–Alop against OSCC based on network pharmacology. (**A**) Venn Diagram of potential target of Wog–Alop with OSCC-related disease target; (**B**) PPI network of the intersection targets between the potential targets of Wog–Alop and the OSCC disease targets; (**C**) Target-pathway network of Wog–Alop against OSCC (the area size and color depth of the node represent its degree value, the larger the area and the darker the color indicate the greater the degree value of this node); (**D**,**E**) GO enrichment and KEGG pathway analysis of Wog–Alop against OSCC.

A total of 284 potential targets for Wog–Alop were predicted by the TCMSP, Swiss, Super Pred, and Pharm Mapper databases; meanwhile, 834 disease targets for OSCC were collected by Genecards and OMIM databases. After that, 49 intersected targets were obtained by intersecting the Wog–Alop potential targets with OSCC disease targets through Draw Venn Diagram (Figure 2A). Intersecting targets were submitted to the String platform to construct the PPI network, which contains 49 nodes representing proteins and 134 edges representing interactions between proteins. The generated “string interactions” file was imported into Cytoscape 3.7.1 for analysis and the PPI network was visualized according to the degree value. The results are shown in Figure 2B, the area of node in the graph represents the degree value of that node, and the larger the node in the graph, the higher the degree value. The visualization results of the target-pathway network are shown in Figure 2C, and based on the node degree values, it could be speculated that PIK3R1, CCND1, CASP3, BCL2, EGFR, TP53, AKT1, BAX, CASP9, and MET might be the key targets of Wog–Alop anti-OSCC.

The above intersected targets were subjected to GO and KEGG analysis (*p* < 0.05) using the Metascape online analysis platform. As shown in Figure 2D, 10 items each of biological processes (BP), cellular component (CC) and molecular function (MF) in GO analysis were selected, involving cell population proliferation, regulation of apoptotic signaling pathway, transcription coregulator binding and Bcl-2 family protein complex. To explore the signaling pathway of Wog–Alop for OSCC treatment, KEGG enrichment analysis was performed. As shown in Figure 2E, the top 20 signaling pathways were displayed, involving pathways in cancer, PI3K-Akt signaling pathway, p53 signaling pathway. The top 20 signaling pathways enriched from KEGG analysis and 49 intersection targets were selected to draw the target-pathway network by Cytoscape 3.7.1 software. 

### 3.3. Molecular Docking and Dynamics Simulations

While pathway enrichment analysis revealed stronger alterations in PI3K-AKT, ROS, and VEGF signaling, therapeutic target selection must additionally consider factors including druggability, selectivity, and clinical translatability. Extensive research demonstrates that PI3K-AKT inhibition induces apoptosis through Bax activation and Bcl-2 downregulation, ROS accumulation triggers mitochondrial apoptosis via Bcl-2 family disruption, and VEGF blockade removes survival signals that maintain the Bcl-2/Bax balance. Therefore, apoptosis-related targets P53, Caspase3, Bax and Bcl-2 were selected as validation targets because they represent the convergence point where these multiple pathways execute their anticancer effects, offering superior druggability and established clinical relevance. Structural selection criteria prioritized templates with co-crystallized ligands in druggable pockets relevant to our mechanistic hypothesis, minimal crystallographic artifacts, and established structure–activity relationships. All co-crystallized ligands and cofactors were retained during initial docking to preserve native binding site conformations, then removed for production runs to avoid steric bias. 

**Figure 3:**
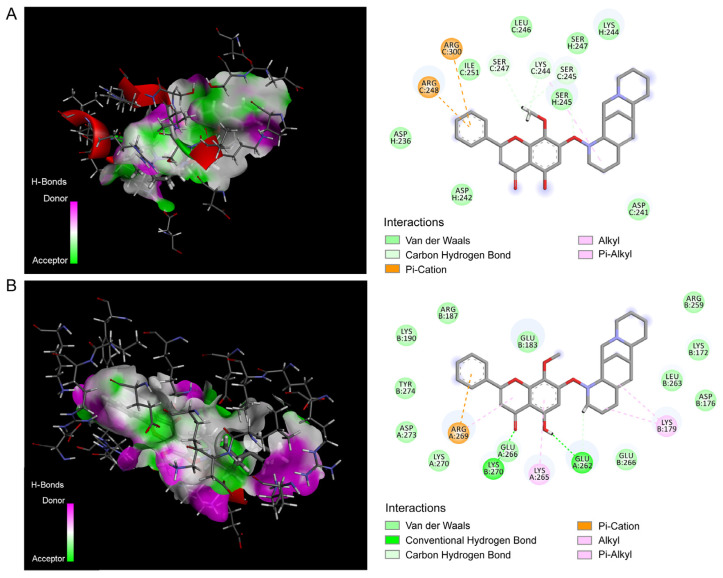
Molecular docking of Wog–Alop with Bax and Bcl-2 and their interaction diagrams. (**A**) Molecular docking of Wog–Alop with Bax (PDB ID: 8eja); (**B**) Molecular docking of Wog–Alop with Bcl-2 (PDB ID: 4zbi).

According to the prediction results (Figure 3), the interaction between wogonin (Figure S2), aloperine (Figure S3)and Wog–Alop with P53, Caspase3, Bax and Bcl-2 targets were further explored by molecular docking. Among them, docking of Caspase3 and P53 with wogonin, aloperine and Wog–Alop molecule was not obtained, and it could be reasonably explained by the fact that there is no direct interaction between Caspase3 and P53 with these ligands, which has also be also been confirmed in the following publication [36]. As shown in Table 1, the “CDOCKER INTERATION ENERGY” between Wog–Alop with Bcl-2 and Bax are 39.4304 and 40.0551 kcal/mol, respectively, which is higher than that of wogonin and aloperine, indicating that the formation of Wog–Alop significantly increased its binding ability to the target protein. 

**Figure 4:**
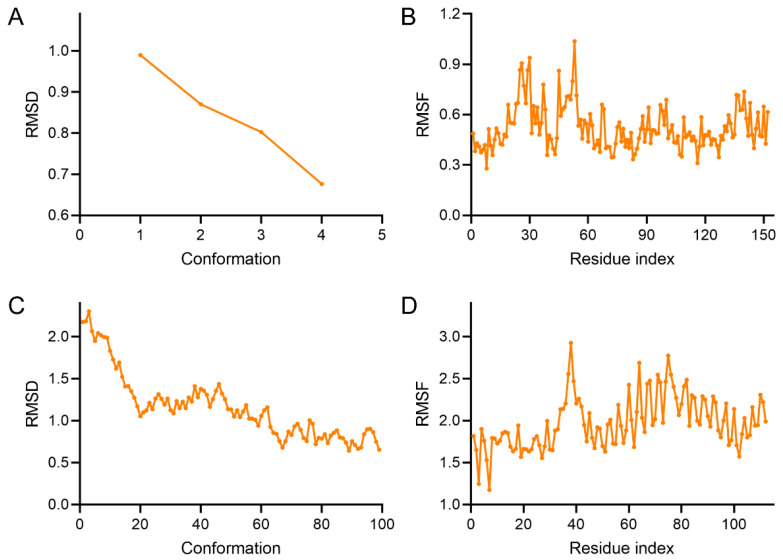
Molecular dynamics simulations. (**A**) RMSD of Wog–Alop–Bcl-2 complex, (**B**) RMSF of Wog–Alop–Bcl-2 complex, (**C**) RMSD of Wog–Alop–Bax complex, (**D**) RMSF of Wog–Alop–Bax complex.

Furthermore, the stability of the protein-ligand complexes formed by Wog–Alop with Bcl-2 and Bax were further explored by molecular dynamics simulations. RMSD and RMSF are important parameters in molecular dynamics, which are commonly used to describe the conformational changes in proteins or molecular ligands as well as the range and intensity of motion of each atom in the protein structure. As shown in Figure 4, where the RMSD and RMSF of Wog–Alop-target complex were small, indicating that the complexes of Wog–Alop with Bcl-2 and Bax are structurally stable, and the binding capacity between Wog–Alop and targets are stronger compared to wogonin (Figure S4), and aloperine (Figure S5). These results suggest that Wog–Alop may realize the anti-OSCC activity by increasing the binding ability between wogonin and core key targets.

### 3.4. Wog–Alop Induced the Apoptosis of Tumor Cells

**Figure 5:**
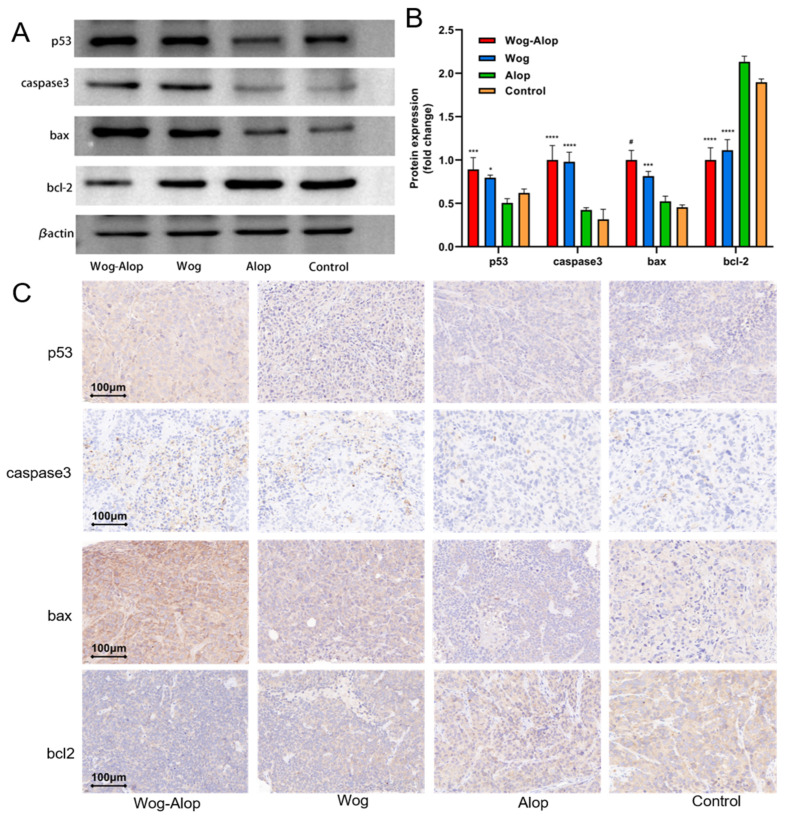
The induction of tumor cell apoptosis in OSCC by Wog, Alop, and Wog–Alop. (**A**) Western blot of the apoptosis-associated protein in OSCC tumor tissues; (**B**) Apoptosis-associated proteins were quantified by Western blot; (C) Representative immunohistochemical images of Bax, Bcl-2, P53, and Caspase3 in OSCC tissues (400×). Data are presented as mean ± SD (*n* = 6); (* *p* < 0.05, *** *p* < 0.001, **** *p* < 0.0001 versus control; # *p* < 0.05 versus Wog group).

The apoptosis of tumor cells can be used to determine in part the effect of anticancer drugs, which is regulated by the Bcl-2 family. Specifically, pro-apoptotic Bax promotes the cytosolic release of cytochrome C and activates one of the key executioners of apoptosis, Caspase-3, leading to apoptosis, while the down-regulation of anti-apoptotic protein Bcl-2 indicates the release of apoptosis suppression in tumor cells [37]. Additionally, P53 is one of the most commonly used tumor markers, which protects against tumor formation by preventing the accumulation of cells with DNA damage. To investigate the apoptotic mechanism, the expression levels of Bcl-2, Bax, P53, and Caspase-3 were examined in OSCC tissues by Western blot (Figure 5A,B). The results demonstrated that Wog–Alop significantly down-regulated the expression of Bcl-2 and up-regulated the expressions of Bax and Caspase-3 compared to wogonin, aloperine, and control groups (Figure 5B). Meanwhile, the upregulation of P53 was observed in the Wog–Alop treatment group, suggesting that Wog–Alop treatment resulted in P53 upregulation concurrent with growth suppression, indicating activation of stress response pathways, though the specific upstream triggers remain to be determined. 

Notably, it could be seen that among the apoptosis-related targets (P53, Caspase3, Bax and Bcl-2), Wog–Alop treatment significantly enhanced the pro-apoptotic protein Bax compared to Wog monotherapy (*p* < 0.05), which is a particularly important finding given Bax’s central role as a mitochondrial gateway for apoptosis execution, representing a targeted therapeutic mechanism that warrants further investigation in larger cohorts. However, statistical comparisons between Wog–Alop and Wog treatment groups revealed significant differences for pro-apoptotic protein Bax, whereas no significant differences were observed for Bcl-2, Caspase-3 and P53. These selective Bax activators have shown therapeutic efficacy through direct mitochondrial engagement rather than broad apoptotic pathway activation [38]. The absence of immediate Caspase-3 activation despite Bax upregulation may reflect the temporal dynamics of apoptotic signaling [39], and our observation timepoint may have captured early mitochondrial signaling before full caspase commitment. The lack of P53 modulation suggest that Wog–Alop operates through P53-independent mechanisms, potentially via direct mitochondrial effects as described for ARTS-mediated pathways [40]. Furthermore, the selective enhancement pattern aligns with current drug conjugation strategies that achieve therapeutic efficacy through targeted pathway modulation rather than broad-spectrum effects. The Alop modification may confer advantages through specific mechanisms such as enhanced mitochondrial targeting or improved cellular uptake, consistent with mitochondria-targeting conjugate strategies.

**Figure 6:**
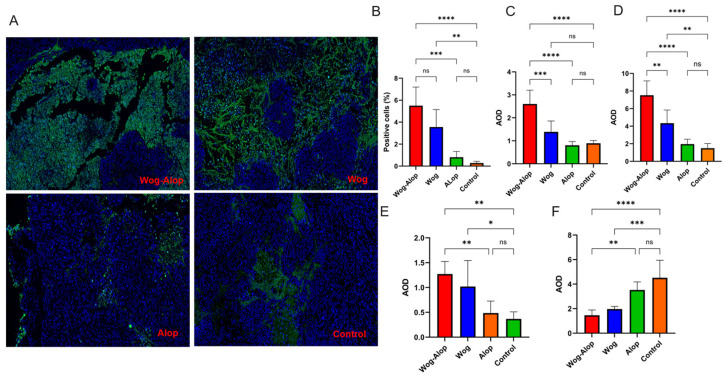
Expression of apoptosis-related proteins in OSCC tumor tissues. (**A**) Representative image showing TUNEL-positive apoptotic nuclei (green) with DAPI nuclear counterstain (blue); (**B**) Quantification analysis of TUNEL-positive apoptotic rates of apoptotic cells; (**C**–**F**) Quantitative analysis of immunohistochemical staining of P53, Caspase3, Bax, and Bcl-2, respectively. Data are presented as mean ± SD (* *p* < 0.05, ** *p* < 0.01, *** *p* < 0.001, **** *p* < 0.0001versus control or Wog group). (*n* = 6).

To further validate the apoptotic effects, immunohistochemical analysis was performed (Figure 5C). The apoptosis of cells in OSCC tissues was directly assessed through TdT-mediated dUTP nick-end labeling (TUNEL) assay (Figure 6A,B). Consistent with the Western blot results, the enhanced expressions of Bax and Caspase-3 and the decreased expression of Bcl-2 were also detected in immunohistochemical staining (Figure 6C–F). Furthermore, the proportion of apoptotic cells in the Wog–Alop group was significantly higher than that in the wogonin and aloperine groups, implying that the improved solubility contributes to better anti-tumor efficacy in OSCC. Overall, these results indicated that the apoptotic mechanism is implicated in Wog–Alop-induced inhibition of proliferation in OSCC.

### 3.5. Evaluation of Multiple Organ Toxicological of Wog–Alop

**Figure 7:**
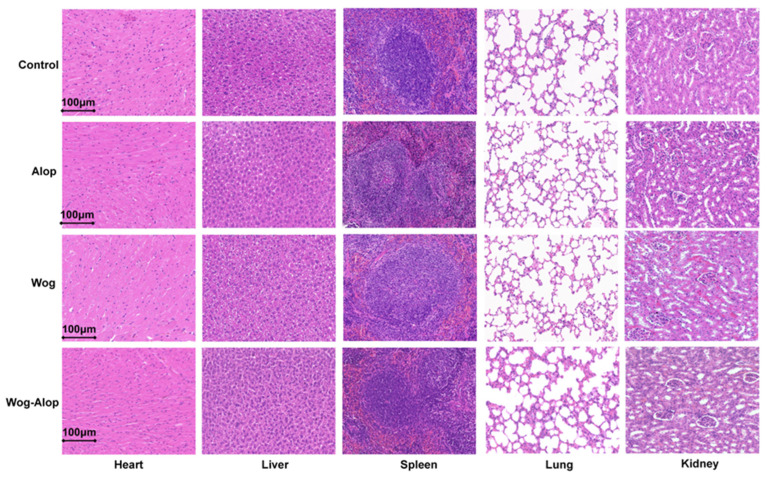
H&E staining(400×) of heart, liver, spleen, lung and kidney removed from the treated control nude mice to evaluate the toxicity of wogonin, aloperine and Wog–Alop.

The histologic sections stained with Hematoxylin and eosin (H&E) were performed to evaluate the toxicity of Wog, Alop and Wog–Alop. As shown in Figure 7, compared with the control group, there is no significant tissue morphology damage in the heart, liver, spleen, lung and kidney treated with Wog, Alop and Wog–Alop, highlighting the superiority of Wog–Alop as a potential antitumor drug. In addition, Masson’s Trichrome and Sirus Red staining was performed on the heart, spleen, lung, kidney and liver tissue removed from the treated and control nude mice, and the Masson’s Trichrome staining results showed no obvious collagen deposition and significant enlargement of the cell gap were observed. Meanwhile, the proportion of collagen type I and collagen III was not decreased after Sirius red staining (Figure S6).

## 4. Discussion

OSCC is a major epithelial malignancy of the head and neck [41], posing significant therapeutic challenges in clinical practice due to complex pathologic subtypes, genomic heterogeneity, and acquired drug resistance. The limitations of current chemotherapeutic regimens are well-documented, with conventional agents exhibiting substantial toxicities including cardiotoxicity and severe gastrointestinal adverse effects, thereby compromising treatment efficacy and patient quality of life. These clinical realities underscore the urgent need for innovative therapeutic strategies with improved selectivity and reduced systemic toxicity.

Natural products demonstrate synergistic enhancement effects in combination therapies and achieve reduction in severe adverse events compared to conventional chemotherapy, which makes them highly efficacious in the treatment of cancer. The present study established Wog–Alop as a promising natural therapeutic candidate for OSCC through systematic validation using PDX model. Compared to existing natural products and traditional chemotherapy agents, Wog–Alop demonstrates clear advantages in multi-target regulation, safety, and potential combination therapy applications. For instance, curcumin primarily functions through the NF-κB signaling pathway [42], while resveratrol mainly regulates the mitochondrial apoptosis pathway [43]. Quercetin exerts anticancer effects by inducing G2/M phase arrest [44]. In contrast, Wog–Alop simultaneously orchestrates Bcl-2/Bax ratio modulation, P53 activation, and caspase-3 cascade initiation, representing a comprehensive apoptotic response that may circumvent single-pathway resistance mechanisms observed in OSCC. Furthermore, Wog–Alop showed no significant toxicity to normal tissues, avoiding the acute kidney injury incidence associated with cisplatin and the cardiotoxicity inherent to 5-fluorouracil therapy [45]. However, our molecular docking simulations suggest potential binding interactions, and protein expression analyses demonstrate consistent modulation of apoptotic markers, but these findings indicate pathway-level regulation rather than confirmed direct target engagement. Future studies employing co-immunoprecipitation, surface plasmon resonance, or enzymatic inhibition assays would be valuable to definitively establish direct protein-drug interactions. Furthermore, the present study demonstrates P53 upregulation following Wog–Alop treatment, but the precise mechanism of P53 activation remains to be elucidated and DNA damage-specific markers (γ-H2AX, phospho-ATM, 53BP1) and assessment of other stress pathways would be necessary to determine whether Wog–Alop’s effects are mediated through genotoxic stress or alternative P53-activating mechanisms. Collectively, the convergence of multi-target engagement, PDX-validated efficacy, and minimal systemic toxicity established foundation for clinical translation of Wog–Alop as an innovative therapeutic agent for OSCC management.

## 5. Conclusions

In conclusion, the significant inhibition of Wog–Alop against OSCC was validated by a PDX model, which simultaneously orchestrates Bcl-2/Bax ratio modulation, P53 activation, and Caspase3 cascade initiation. Compared to existing natural products and conventional chemotherapeutics, Wog–Alop demonstrates advantages in multi-target regulation, safety, and potential combination therapy applications. These findings contribute to the growing body of evidence supporting natural product-based drug development and provide a paradigm for systematic evaluation of novel anticancer agents. 

## Figures and Tables

**Table 1:** The binding energy between Wog, Alop, and Wog–Alop with key targets.

Target	Ligands	CDOCKER Interaction Energy (kcal/mol)
Bcl-2	Wog–Alop	39.4304
	Wogonin	26.0210
	Aloperine	16.3727
	Wog–Alop	40.0551
BAX	Wogonin	33.5262
	Aloperine	17.7115

## Data Availability

The data used to support the findings of the study are available upon reasonable request from the corresponding author.

## Supplementary Materials

The following supporting information can be downloaded at: https://www.mdpi.com/article/10.3390/pharmaceutics17091204/s1, Figure S1: Body weight changes in nude mice following treatment with Wog-Alop, Alop, Wog, and Control over the treatment period (mean ± SD, n = 6); Figure S2: Molecular docking and molecular of wogonin with Bax and Bcl-2. (A) Molecular docking of wogonin with Bcl-2; (B) Molecular docking of wogonin with Bax; Figure S3: Molecular docking and molecular of aloperine with Bax and Bcl-2. (A) Molecular docking of aloperine with Bcl-2; (B) Molecular docking of aloperine with Bax; Figure S4: Molecular dynamics simulations. (A) RMSD of wogonin-Bcl-2 complex, (B) RMSF of wogonin -Bcl-2 complex, (C) RMSD of wogonin-Bax complex, (D) RMSD of wogonin- Bax complex; Figure S5: Molecular dynamics simulations. (A) RMSD of aloperine-Bcl-2 complex, (B) RMSF of aloperine-Bcl-2 complex, (C) RMSD of aloperine- Bax complex, (D) RMSD of aloperine- Bax complex; Figure S6: Body weight changes in nude mice following treatment with Wog-Alop, Alop, Wog, and Control over the treatment period (mean ± SD, n = 6); Table S1: The antibodies used for immunohistochemistry staining.

## Abbreviations

The following abbreviations are used in this manuscript:

OSCC	Oral squamous cell carcinoma
Wog	Wogonin
Wog+Alop	Physical mixture of wogonin and aloperine
Wog-Alop	Wogonin-aloperine co-amorphous
TdT	Terminal deoxynucleotidyl transferase
TUNEL	TdT-mediated dUTP nick-end labeling
H&E	Hematoxylin and eosin
CC	Cellular component
BP	Biological processes
BCL2	B-cell lymphoma 2
BAX	B-cell lymphoma 2 associated X protein
RMSF	Root means square fluctuation
RMSD	Root means square deviation
TCMSP	Traditional Chinese Medicine Systems Pharmacology
