# Supplementary material for: Therapeutic Potential of Wogonin–Aloperine Co-Amorphous for Oral Squamous Cell Carcinoma
Source: Pharmaceutics. 2025 Sep 16;17(9):1204. doi: 10.3390/pharmaceutics17091204 (PMC12473305; doi:10.3390/pharmaceutics17091204)
Supplement: Supplementary file 1 [file pharmaceutics-17-01204-s001.zip › pharmaceutics-3806252-supplementary.pdf]

pharmaceutics

Supplementary Material

Therapeutic Potential of Wogonin–Aloperine Co-Amorphous for Oral Squamous Cell Carcinoma

Guoliang Wu [2,†], Han Li [2,†], Zhongshui Xie [1], Song Ni [2], Yiming Zhu [2], Chunxue Jia [1], Chenyu Pan [1], Shaoyan Liu [2,*] and Hongjuan Wang [1,*]

[1] School of Chinese Materia Medica, Beijing University of Chinese Medicine, Beijing 100102, China; 15735010317@163.com (Z.X.); a15242876702@163.com (C.J.); xbpcyatx@163.com (C.P.)
[2] Department of Head and Neck Surgery, National Cancer Center/National Clinical Research Center for Cancer/Cancer Hospital, Chinese Academy of Medical Sciences and Peking Union Medical College, Beijing 10029, China; wugl_cicams@outlook.com (G.W.); lihanw11@163.com (H.L.); nisong168@sina.com (S.N.); drymzhu@163.com (Y.Z.)
* Correspondence: liushaoyan@cicams.ac.cn (S.L.); wang1226a@bucm.edu.cn (H.W.)
† These authors contributed equally to this work.

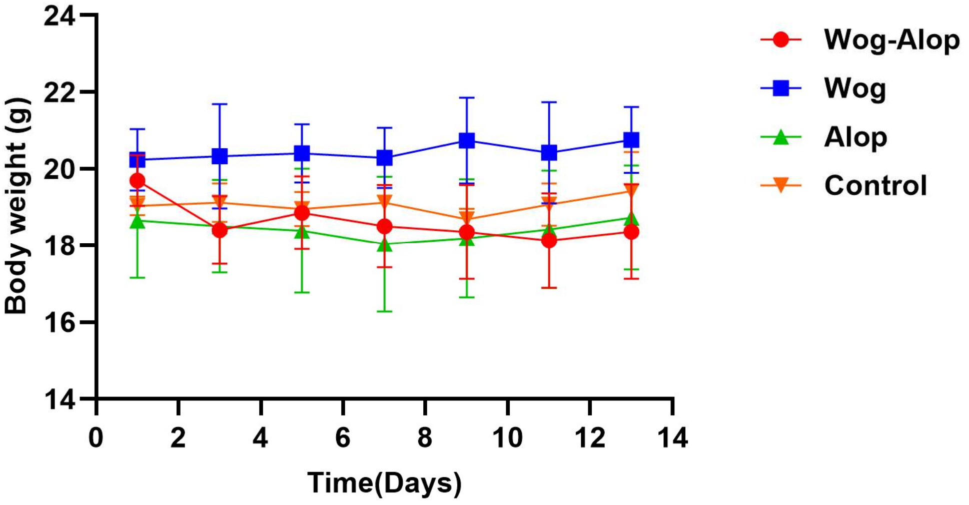

Figure S1. Body weight changes in nude mice following treatment with Wog-Alop, Alop, Wog, and Control over the treatment period (mean ± SD, *n* = 6).

Academic Editors: Dimitrios A. Lamprou and Juan Torrado

Received: 23 July 2025
Revised: 29 August 2025
Accepted: 5 September 2025
Published: 16 September 2025

Citation: Wu, G.; Li, H.; Xie, Z.; Ni, S.; Zhu, Y.; Jia, C.; Pan, C.; Liu, S.; Wang, H. Therapeutic Potential of Wogonin–Aloperine Co-Amorphous for Oral Squamous Cell Carcinoma. *Pharmaceutics* **2025**, *17*, 1204. https://doi.org/10.3390/pharmaceutics17091204

Copyright: © 2025 by the authors. Licensee MDPI, Basel, Switzerland. This article is an open access article distributed under the terms and conditions of the Creative Commons Attribution (CC BY) license (https://creativecommons.org/licenses/by/4.0/).

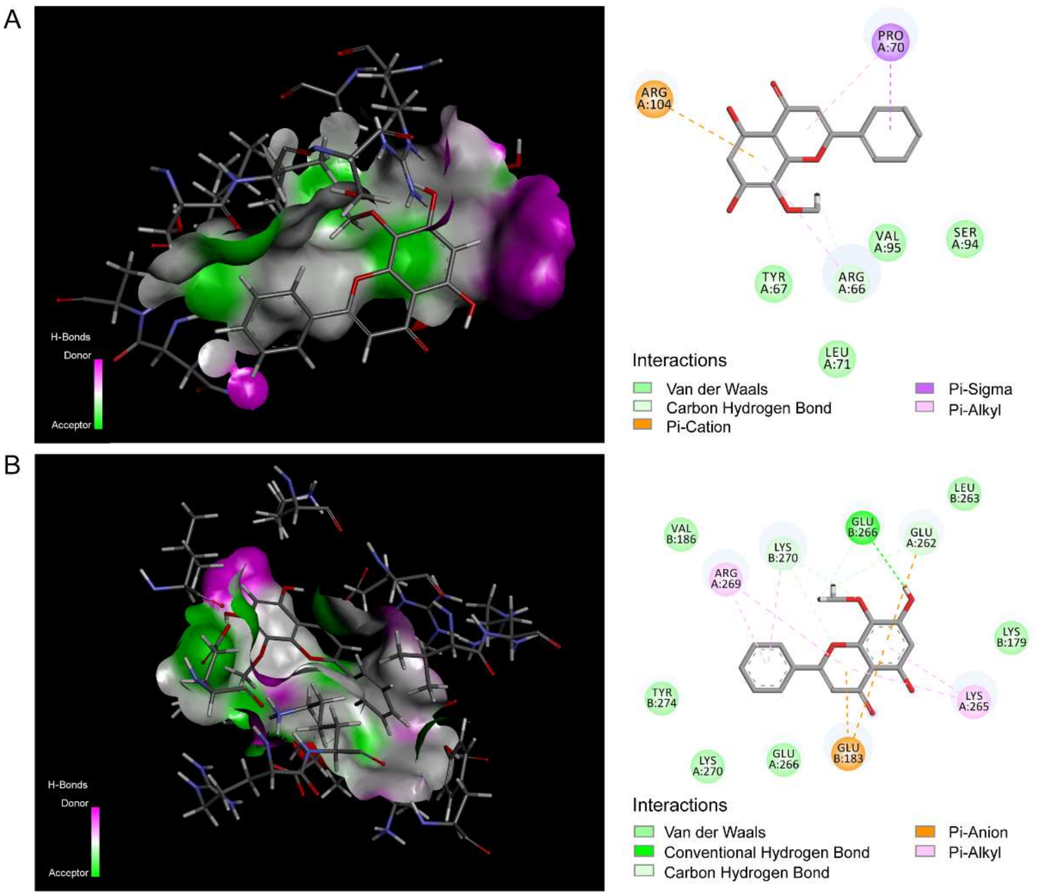

Figure S2. Molecular docking and molecular of wogonin with Bax and Bcl-2. (A) Molecular docking of wogonin with Bcl-2; (B) Molecular docking of wogonin with Bax.

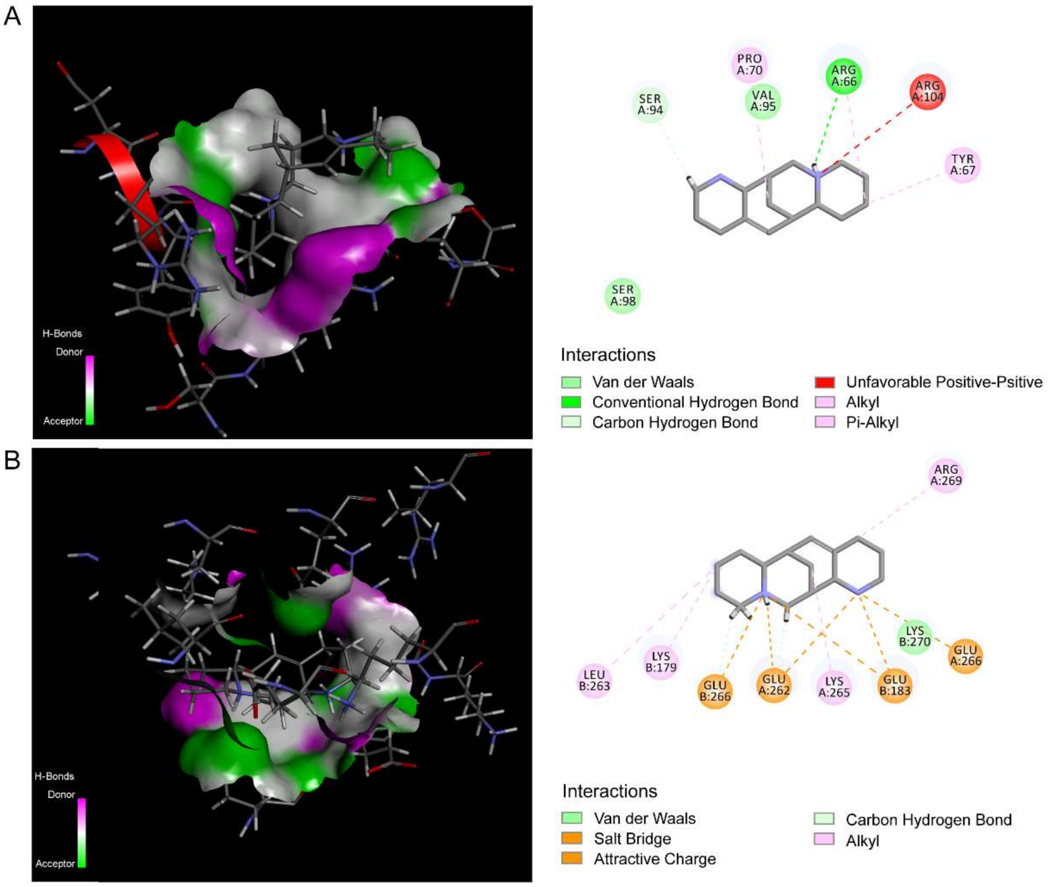

Figure S3. Molecular docking and molecular of aloperine with Bax and Bcl-2. (A) Molecular docking of aloperine with Bcl-2; (B) Molecular docking of aloperine with Bax.

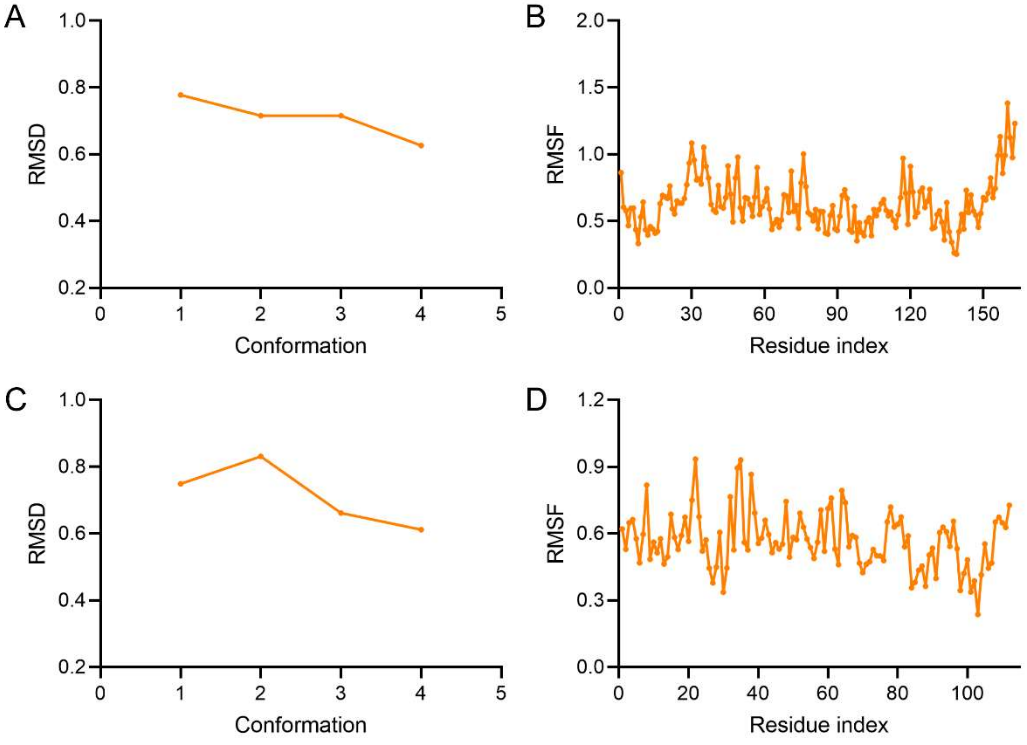

Figure S4. Molecular dynamics simulations. (A) RMSD of wogonin-Bcl-2 complex, (B) RMSF of wogonin -Bcl-2 complex, (C) RMSD of wogonin-Bax complex, (D) RMSD of wogonin- Bax complex.

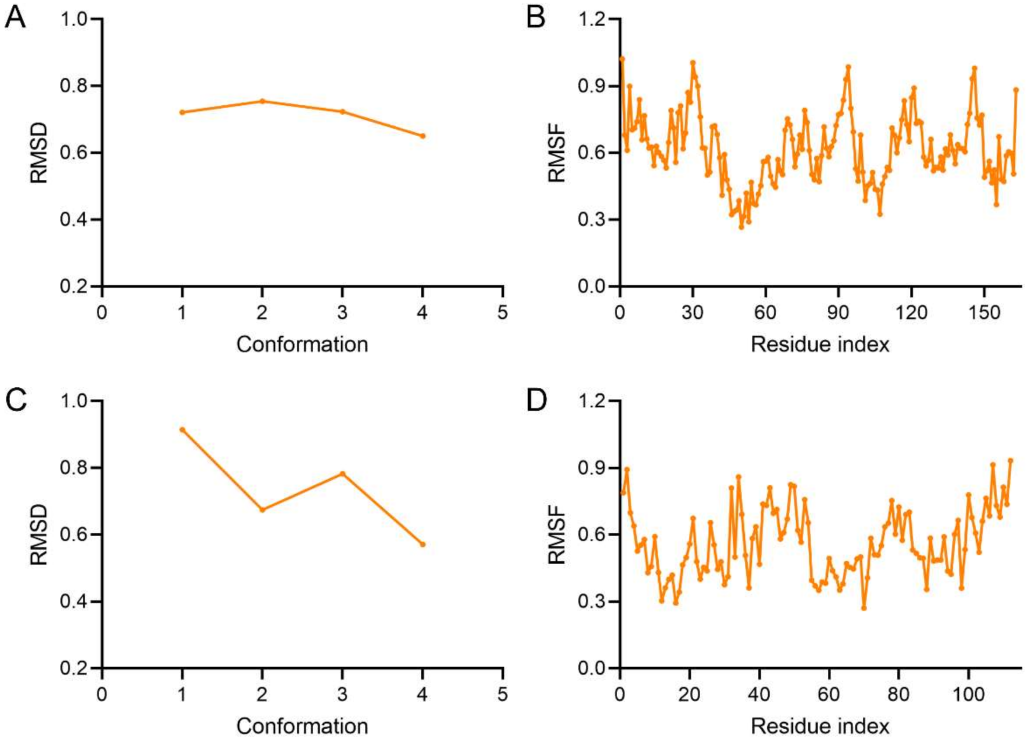

Figure S5. Molecular dynamics simulations. (A) RMSD of aloperine-Bcl-2 complex, (B) RMSF of aloperine-Bcl-2 complex, (C) RMSD of aloperine- Bax complex, (D) RMSD of aloperine- Bax complex.

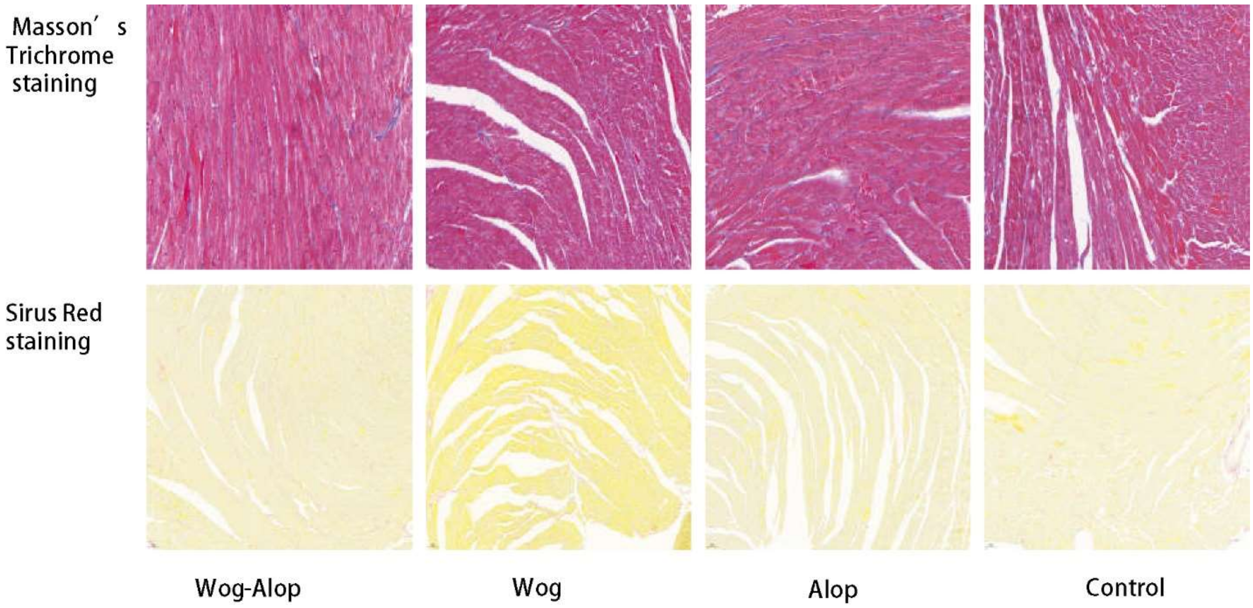

Figure S6. Masson's Trichrome and Sirus Red staining (400x) of heart, liver, spleen, lung and kidney removed from the treated and control mice to evaluate the toxicity of Alop, Wog and Wog-Alop.

Table S1. The antibodies used for immunohistochemistry staining.

Secondary antibody	Item No.	Manufacturer	Dilution ratio
HRP labeled rabbit anti-goat IgG	GB23204	Servicebio	1:200
HRP labeled goat anti-mouse IgG	GB23301	Servicebio	1:200
HRP labeled goat anti-rat IgG	GB23302	Servicebio	1:200
HRP labeled goat anti-rabbit secondary antibody	GB23303	Servicebio	1:200
HRP labeled donkey anti-goat secondary antibody	GB23404	Servicebio	1:200
